# High Yield Production of Influenza Virus in Madin Darby Canine Kidney (MDCK) Cells with Stable Knockdown of IRF7

**DOI:** 10.1371/journal.pone.0059892

**Published:** 2013-03-26

**Authors:** Itsuki Hamamoto, Hiroshi Takaku, Masato Tashiro, Norio Yamamoto

**Affiliations:** 1 Laboratory of Cell-based Vaccine Development, Influenza Virus Research Center, National Institute of Infectious Diseases, Musashimurayama-shi, Tokyo, Japan; 2 Department of Life and Environmental Science, Chiba Institute of Technology, Narashino-shi, Chiba, Japan; 3 Department of General Medicine, Juntendo University School of Medicine, Bunkyo-ku, Tokyo, Japan; The University of Hong Kong, China

## Abstract

Influenza is a serious public health problem that causes a contagious respiratory disease. Vaccination is the most effective strategy to reduce transmission and prevent influenza. In recent years, cell-based vaccines have been developed with continuous cell lines such as Madin-Darby canine kidney (MDCK) and Vero. However, wild-type influenza and egg-based vaccine seed viruses will not grow efficiently in these cell lines. Therefore, improvement of virus growth is strongly required for development of vaccine seed viruses and cell-based influenza vaccine production. The aim of our research is to develop novel MDCK cells supporting highly efficient propagation of influenza virus in order to expand the capacity of vaccine production. In this study, we screened a human siRNA library that involves 78 target molecules relating to three major type I interferon (IFN) pathways to identify genes that when knocked down by siRNA lead to enhanced production of influenza virus A/Puerto Rico/8/1934 in A549 cells. The siRNAs targeting 23 candidate genes were selected to undergo a second screening pass in MDCK cells. We examined the effects of knockdown of target genes on the viral production using newly designed siRNAs based on sequence analyses. Knockdown of the expression of a canine gene corresponding to human IRF7 by siRNA increased the efficiency of viral production in MDCK cells through an unknown process that includes the mechanisms other than inhibition of IFN-α/β induction. Furthermore, the viral yield greatly increased in MDCK cells stably transduced with the lentiviral vector for expression of short hairpin RNA against IRF7 compared with that in control MDCK cells. Therefore, we propose that modified MDCK cells with lower expression level of IRF7 could be useful not only for increasing the capacity of vaccine production but also facilitating the process of seed virus isolation from clinical specimens for manufacturing of vaccines.

## Introduction

Influenza is a global public health issue that causes a serious illness with a high mortality rate. Vaccination is one of the most effective medical strategies to prevent influenza virus infection. The current egg-based technology for manufacturing influenza vaccine has been used since 1950s, but cell-based technology has been developed to produce more effective influenza vaccines in sufficient quantities in a shorter period of time. In recent years, two continuous cell lines have been approved by regulatory authorities to be used for the production of influenza vaccines: Madin Darby canine kidney (MDCK) cells and African green monkey kidney-derived Vero cells [1]–[5]. Human retina-derived cell line PER.C6 has also been shown useful for propagation of influenza viruses [6]. Although these cell lines produce notable yields of a wide variety of influenza viruses, attempts to develop novel cell lines with greater potentials have been made for more rapid preparation of influenza vaccines. A recent study demonstrated that the *siat7e*-expressing MDCK cells produce much more HA antigen than the parental MDCK cells [5]. The *siat7e*-expressing cells that proliferate in suspension would facilitate influenza virus productions [5].

Influenza viruses have been shown to be recognized by pattern-recognition receptors (PRRs), such as the Toll-like receptors 3 (TLR3) [7] and 7/8 (TLR7/8) [8], and retinoic acid-inducible gene (RIG-I)-like receptors (RLRs) [9]. Influenza viruses have evolved strategies to counteract cellular antiviral mechanisms, especially to circumvent the type I interferon (IFN-α/β) system which is a first line of defense against viral infections [10], [11]. The mechanisms that underlie the induction of type I IFN genes have been extensively studied in the context of immunity to viruses. Type I IFNs are known to be critical for inflammation [12] and play central roles in the activation of host antiviral responses to control virus infections [13], [14]. Therefore, we hypothesized that it would be possible to obtain novel MDCK cells with greater potential to propagate influenza viruses by means of RNA-interference to inhibit the function of genes relating to IFN signaling.

In this study, we identified canine IRF7 as a target in order to establish modified MDCK cells which would be capable of producing higher yield of influenza A viruses. Knockdown of IRF7 with siRNA showed 3 to 4-fold enhancement of influenza A virus production. We also confirmed that MDCK cells with stable knockdown of IRF7 by short hairpin RNA (shRNA) showed 2 to 8-fold enhancement of influenza virus production. In conclusion, the modified MDCK cells with lower level of IRF7 expression may be useful for producing higher titers of influenza viruses. The novel MDCK cells will be beneficial for large-scale production of influenza vaccines in the manufacturing process. In addition, the established MDCK cells will be useful for isolation of influenza viruses from clinical specimens with a low viral load and efficient preparation of vaccine seed viruses in a shorter period.

## Materials and Methods

### Cells

A549 cells were purchased from Japanese Collection of Research Bioresources (JCRB; Osaka Japan). A549 cells were cultured in Dulbecco’s modified Eagle’s medium (DMEM; GIBCO, Carlsbad, CA) containing 10% fetal bovine serum (FBS; GIBCO, Carlsbad, CA) and 100 units/ml penicillin/streptomycin (GIBCO, Carlsbad, CA). MDCK (CCL-34) cells were purchased from American Type Culture Collection (ATCC; Rockville, MD). MDCK cells were cultured in Opti-Pro serum free medium (Opti-pro SFM; GIBCO, Carlsbad, CA) containing 4 mM L-Glutamine (GIBCO, Carlsbad, CA). All cells were cultured in a 5% CO_2_ humidified incubator.

### Viruses

Viruses used in this study were A/Puerto Rico/8/1934 (PR8; A(H1N1)), A/Narita/1/2009 (NR1; A(H1N1)pdm09), A/Victoria/361/2011 (VC361; A(H3N2)), and B/Florida/4/2006 (FL4; type B, Yamagata lineage). These viruses were propagated in MDCK cells. The virus titers were determined by 50% tissue culture infectious dose (TCID_50_) assay or plaque assay.

### siRNA Transfection, Virus Infection and RNA Extraction

The siRNA duplexes for A549 cells were selected from Silencer select siRNA library (Ambion, Austin, TX). The siRNA duplexes for MDCK cells were synthesized (Sigma-Aldrich, St. Louis, MO). The sequences of siRNA duplexes for MDCK cells were listed in Table 1. A549 cells were seeded on type I collagen coated 96-well plate. Three target-specific siRNAs (Silencer select siRNA library, Ambion, Austin, TX) or non-targeting negative control siRNA (Silencer Negative Control #1 siRNA, Ambion, Austin, TX) at a final concentration of 10 nM were transfected using transfection reagent (Lipofectamine RNAiMAX; Invitrogen, Carlsbad, CA) and incubated at 37°C for 48 h. For MDCK cells, the cells were seeded on 96-well plate and three target-specific siRNAs (Sigma-Aldrich, Inc., St. Louis, MO) or non-targeting negative control siRNA (Silencer Negative Control #1 siRNA, Ambion, Austin, TX) at a final concentration of 50 nM were transfected by siRNA transfection reagent (Lullaby; OZ Biosciences, France). Cells were washed with PBS three times and infected with influenza A virus at a MOI of 0.01 in Opti-Pro SFM in the presence of 2 µg/ml of trypsin acetylated (Sigma, Chemical Co., St. Louis, MO) at 34°C for 1 h. Then, cells were washed with PBS three times and incubated in Opti-Pro SFM supplemented with 2 µg/ml of trypsin acetylated at 37°C for 24 h. The viral RNA from tissue culture supernatant was mixed with the lysis buffer containing carrier RNA derived from uninfected A549 or MDCK cells and was extracted using MagMAX™-96 Blood RNA Isolation Kit (Ambion, Austin, TX) on King Fisher purification systems (Thermo Scientific, Cambridge, MA). The total RNA from cultured cells was extracted using RNeasy Mini Kit (Qiagen, Hilden, Germany) followed by DNase I (Qiagen, Hilden, Germany) treatment.

**Table 1:** Canis lupus familiaris siRNA used in this study.

Target Gene	Orientation	Sequences (5′ to 3′)
IRF7_1	Sense	CUGGGCAAAUGCAAGGUCUTT
IRF7_1	Anti-sense	AGACCUUGCAUUUGCCCAGTT
IRF7_2	Sense	GGCGCCUGGGCAAAUGCAATT
IRF7_2	Anti-sense	UUGCAUUUGCCCAGGCGCCTT
IRF7_3	Sense	CAGAGAAGCUGCUGCAGCATT
IRF7_3	Anti-sense	UGCUGCAGCAGCUUCUCUGTT
IRF3_1	Sense	GAUCUGAUUGCCUUCAUCATT
IRF3_1	Anti-sense	UGAUGAAGGCAAUCAGAUCTT
IRF3_2	Sense	GGCUCUUGGUGCCUGAUGATT
IRF3_2	Anti-sense	UCAUCAGGCACCAAGAGCCTT
IRF3_3	Sense	CAGACAGUCUCCUGCCCAATT
IRF3_3	Anti-sense	UUGGGCAGGAGACUGUCUGTT
MyD88_1	Sense	GGGCAAAUGCCUGAGCGUUTT
MyD88_1	Anti-sense	AACGCUCAGGCAUUUGCCCTT
MyD88_2	Sense	CAGACAAACUAUCGGCUGATT
MyD88_2	Anti-sense	UCAGCCGAUAGUUUGUCUGTT
MyD88_3	Sense	GCAUCACCAUGCUUGAUGATT
MyD88_3	Anti-sense	UCAUCAAGCAUGGUGAUGCTT
DDX58_1	Sense	CAAACUGUGUGCUUCUCUUTT
DDX58_1	Anti-sense	AAGAGAAGCACACAGUUUGTT
DDX58_2	Sense	GUGUUUCAGUUACCCAACATT
DDX58_2	Anti-sense	UGUUGGGUAACUGAAACACTT
DDX58_3	Sense	GAUCUGAUUGCCUUCAUCATT
DDX58_3	Anti-sense	UGAUGCAUUUAAAUCUGUCTT

### Plasmids

The pRetro-U6 vector was constructed by inserting a human U6 promoter amplified by PCR from genomic DNA into pMSCV-puro (Clontech, Mountain View, CA), from which DNA sequence between Nhe I and Xba I in the 3' LTR was deleted for generation of a self-inactivating virus. DNA fragments encoding the small hairpin RNAs were generated by PCR, digested with Bpi I, and ligated into pRetro-U6 between Bpi I sites downstream of the U6 promoter. The target sequences of shRNAs were as follows: shIRF7, 5'-CTGGGCAAATGCAAGGTCT-3'; shCtrl, 5'-GACTACACAAATCAGCGAT-3' (shCtrl targets LacZ). The shRNA expression cassettes were then transferred to pCS-BS, carrying a blasticidin S resistance gene expressed under the control of the elongation factor 1α promoter. The pCS-BS vector was constructed by replacing EGFP of the pCS-CDF-EG-PRE vector (a kind gift from Dr. Hiroyuki Miyoshi, RIKEN, Tsukuba) with blasticidin S resistance gene amplified by PCR from pcDNA6/myc-His A (Life Technologies, Carlsbad, CA).

### Transduction of MDCK Cells with Lentiviral Vectors

For production of lentiviruses, 293T cells were cotransfected with pCS-BS-shCtrl, or pCS-BS-shIRF7 together with the pCAG-HIVgp, pRSV-Rev (kind gifts from Dr. H. Miyoshi, RIKEN, Tsukuba) and pVSV-G (Clontech, Mountain View, CA) using FuGENE 6 (Roche Applied Science, Indianapolis, IN). Culture supernatants were collected 48 h after transfection and filtered. MDCK cells were transduced with these lentiviruses for 12 h in the presence of 8 µg/mL polybrene and cultured with fresh media. After 48 h of culture, the media were replaced with the selection media containing 10 µg/mL blastcidin S.

### Quantitative Real-time One-step RT-RCR

Real-time RT-PCR reactions were carried out using TaqMan One-step RT-PCR Master Mix Reagents Kit (Applied Biosystems, Foster City, CA) according to the manufacture’s instructions with a total volume of 25 µl. The primers and probes used for quantification of target mRNA were shown in Table 2. The total volume of 5 µl of sample RNA was added into 20 µl of reaction mix. Thermal cycling was performed in a Light Cycler 480 Real-time PCR system II (Roche Diagnostics, Germany) with conditions at 48°C for 30 min, 95°C for 10 min followed by 40 cycles at 95°C for 5 sec and 60°C for 1 min. The amount of target RNA was normalized with the amount of 18S rRNA from host cells or carrier RNA. The knockdown effect on the targeted gene by the specific siRNA was examined by real-time RT-PCR with power SYBR green PCR master mix (Applied Biosystems, Warrington, UK).

**Table 2:** Primer pairs and probes used in this study.

Application	Host	Target	Orientation	Sequence (5′ to 3′)
TaqMan	Homo sapiens	18S	Sense	GTAACCCGTTGAACCCCATT
			Anti-sense	CCATCCAATCGGTAGTAGCG
			Probe	GTGCGTTGATTAAGTCCCTGCCCTTTGTA
	Homo sapiens	IRF7	Sense	CAGAGTCTTCTTCCAAGAGCTGG
			Anti-sense	TGCGTGCCCTCTAGGTGC
			Probe	CCAGGGTTCCAGCTTCACCAGGACC
	Homo sapiens	IRF3	Sense	GCTCGTGATGGTCAAGGTTGTG
			Anti-sense	GAGTGGGTGGCTGTTGGAAATG
			Probe	CCACAGTATTCTCCAGGGAGGAGGCACC
	Canis lupus familiaris	18S	Sense	GCCCGAAGCGTTTACTTTGAAA
			Anti-sense	ATGGCCTCAGTTCCGAAAACC
			Probe	CGAGCCGCCTGGATACCGCAGC
		IRF7	Sense	TCAGCACGTTCTTCCGAGAGAT
			Anti-sense	TAGATGGTGTAGTATGGGGAGCC
			Probe	CCGGCGAGCCCGGAACTCGG
		IRF3	Sense	ACCACGCTACACCCTCTGG
			Anti-sense	CCCTCTAACCGTGCCATTTCC
			Probe	CGTGGGAACAACCTTGAGCATCACCAGC
	Influenza A virus (PR8)	M	Sense	TGCACTTTGACATTGTGGATTCTTG
			Anti-sense	CCCTCATAGACTTTGGCACTCC
			Probe	GCCGTAGAAGGCCCTCCTTTCAGTCCG
	Influenza A virus (NR1)	M	Sense	CACCTGATATTGTGGATTACTGATCG
			Anti-sense	CACTCTGCTGTTCCTGTTGATATTC
			Probe	CCTCATGGACTCAGGCACTCCTTCCG
	Homo sapiens	IFNA	Sense	GGTCACGCTTTCATGAATTCTGT
			Anti-sense	GTGTAAAGGTGCACATGACGTTA
			Probe	TCACCCCTGCTATAACTATGACCATGCTGA
	Homo sapiens	IFNB	Sense	GCTACAACTTGCTTGGATTCCTAC
			Anti-sense	TCCTGTCCTTGAGGCAGTATTC
			Probe	AGCCTCCCATTCAATTGCCACAGGAGC
	Canis lupus familiaris	IFNA	Sense	GCTCTTGTGACCACTACACCA
			Anti-sense	AAGACCTTCTGGGTCATCACG
			Probe	CGCCTCCTGGAGCCGCTGGC
	Canis lupus familiaris	IFNB	Sense	GGATGGAATGAGACCACTGTCG
			Anti-sense	ACGTCCTCCAGGATTATCTCCA
			Probe	GTTCCTTCTGCCAGTGGAGCTTCACAA
PCR	Canis lupus familiaris	IRF7	Sense	ATGGAGCCATACCAGCCACG
			Anti-sense	GGCTCTACCTCCATGAGGAAGT
SYBR	Canis lupus familiaris	IRF7	Sense	TCAGCACGTTCTTCCGAGAGAT
			Anti-sense	TAGATGGTGTAGTATGGGGAGCC
	Canis lupus familiaris	IRF3	Sense	CAGTACCTCGGATACCCAGGAAG
			Anti-sense	GAATGGGGTCAAGACCATGTCAC
	Canis lupus familiaris	MyD88	Sense	GCTTGATGATCCCTCAGGGCAAA
			Anti-sense	CGCTGGGGCAGTAGCAGAT
	Canis lupus familiaris	DDX58	Sense	CAGAATGATCCAAACCAGAGGCA
			Anti-sense	GTTAGAAGGAAGCACTTGCTACC

### Reverse Transcription, Amplification, and Sequence Analysis of mRNA Expressed in MDCK Cells

Reverse transcription was performed using ReverTra-Ace (Toyobo, Osaka, Japan) with random hexamers and the thermal profiles consisted of one cycle at 37°C for 30 min, 42°C for 20 min, 99°C for 5 min and 4°C for 5 min. For detecting a canis lupus familiaris gene corresponding to human IRF7, the cDNA was amplified by KOD Dash polymerase (Toyobo, Japan) using primers shown in Table 2. Thermal cycling conditions were as follows: 30 cycles of 98°C for 10 sec, 55°C for 2 sec and 74°C for 5 min. PCR products were separated by electrophoresis at 100 V for 1 h in a 1.5% (wt/vol) agarose S (Nippon Gene, Tokyo, Japan) gel with TBE buffer and visualized by GelRed Nucleic Acid Gel Stain (Wako, Japan) under UV transillumination. The desired PCR products were extracted and purified using QIAquick gel extraction Kit (Qiagen, Hilden, Germany). The purified products were used for the reaction with BigDye Terminator cycle sequencing ready reaction kit version 3.0 (Applied Biosystems, Foster City, CA). Sequencing alignment was analyzed by using the software Sequencher 4.10.1 (Gene Codes, Ann Arbor, MI).

### Network Analysis

The molecular interaction networks were analyzed by Ingenuity Pathway Analysis (IPA; Ingenuity Systems, Mountain View, CA).

### Virus Infection and Hemagglutination (HA) Assay

The MDCK cells expressing shRNA for IRF7 or control were seeded on a 6-well plate at a density of 2×10^5^ cells/well and cultured until confluent layers were obtained in Opti-pro SFM containing 10 µg/ml of blasticidin S. Cells were infected with indicated amount of PR8 (A/H1N1), NR1 (A/H1N1pdm09), VIC361 (A/H3N2), or FL4 (B/Yam) in Opti-Pro SFM at 34°C for 1 h. Then, cells were cultured with Opti-Pro SFM in the presence of 2 µg/ml of trypsin acetylated and incubated at 37°C for 48–96 h. The culture supernatants were collected for following HA assay. Red blood cells were purchased from Nippon Bio-Test Laboratories, Tokyo, Japan. Guinea pig red blood cells (GRBC) were washed three times with PBS and suspended in PBS at a final concentration of 1% as a working suspension. Turkey red blood cells (TRBC) or chicken red blood cells (CRBC) were washed with 0.85% NaCl and suspended in PBS at a final concentration of 0.5% as a working suspension. For NR1, the virus samples were diluted with PBS serially in U-bottom 96-well plates and then 50 µl of 0.5% TRBC suspension was added into each well and allowed to stand for 45 min at room temperature. For PR8 or VIC361, 50 µl of 1% GRBC suspension was added to serially diluted virus samples and allowed to stand for 60 min at 4°C. For FL4, 50 µl of 0.5% CRBC suspension was added to serially diluted virus samples and allowed to stand for 45 min at room temperature. The HA titer was determined as the highest dilution of the sample showing complete agglutination pattern on the bottom of the well.

## Results

### First siRNA Library Screening

**Figure 1:**
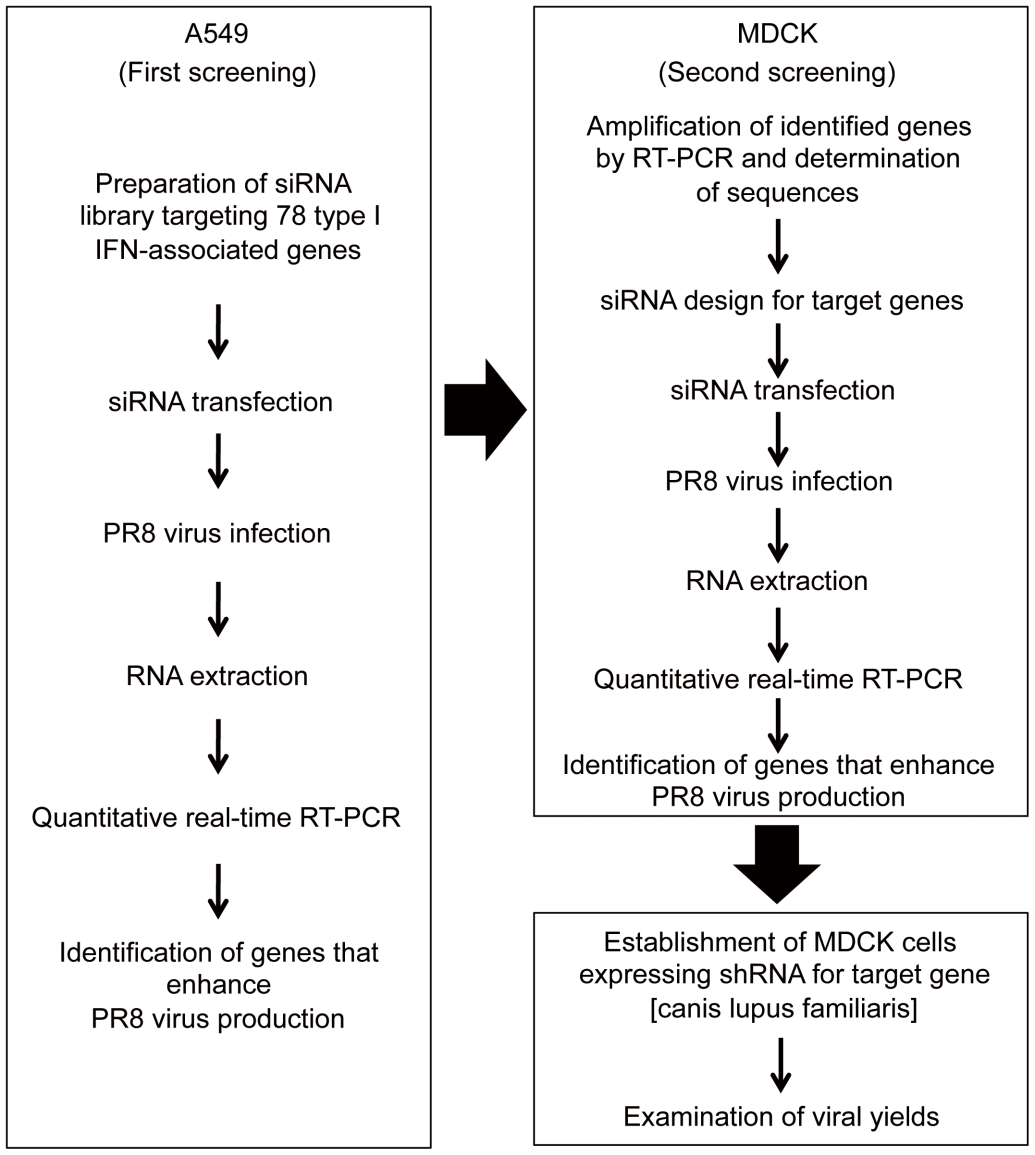
Overview of the study design for the establishment of modified MDCK cells.

The strategy to establish modified MDCK cells with higher efficiency of influenza virus propagation was summarized in the flowchart (Fig. 1). To identify key host factors in canine MDCK cells, we used A549 human lung adenocarcinoma cell line for the first screening because human genome database includes much more information than canis lupus familiaris genome database and related resources including pre-designed siRNA library are available in human.

We screened 78 human genes associated with type I IFN using siRNA library, for enhancement of the propagation of influenza A virus in A549 cells. The siRNAs for each target gene were individually transfected into A549 cells, and the cells were infected with influenza A/Puerto Rico/34/8 (PR8) at a MOI of 0.01. We used PR8 virus, because PR8 has suitable growth properties in many cell lines and is widely used as a backbone virus for the development of high growth reassortants for vaccine production [15]. In the first screening, when knockdown of a cellular gene by the corresponding siRNA showed more than 2-fold enhancement of virus propagation, the gene was considered positive (Fig. S1). A total of 23 out of 78 genes were chosen for further analysis to validate the positive effect of siRNA in influenza virus multiplication.

### Knockdown of IRF7 Enhances the Production of PR8 Virus in A549 Cells

**Figure 2:**
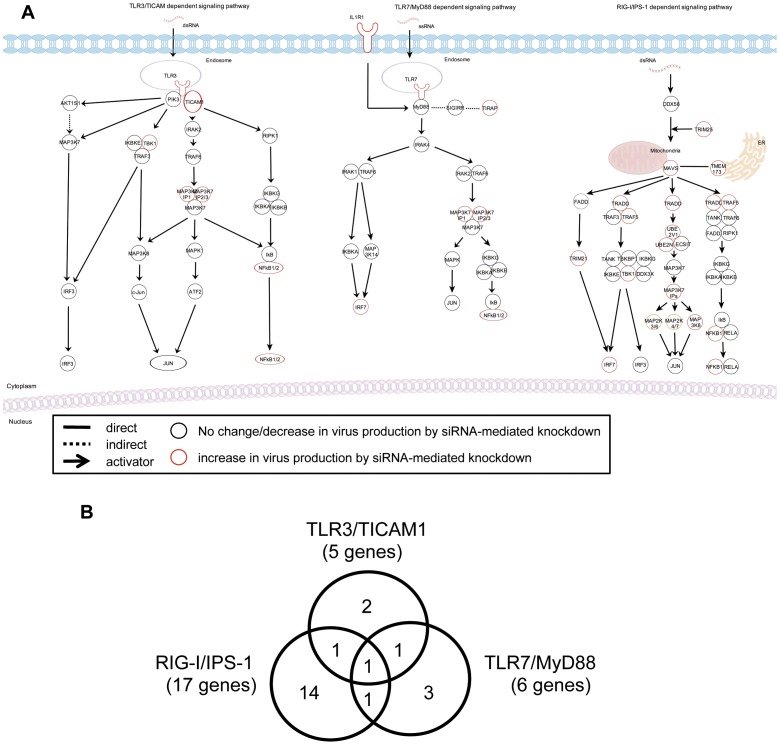
Mapping of the hit genes in the main pathways regarding type I IFN. (A) Ingenuity Pathway Analysis (IPA) was employed to map hit genes that when knocked down by siRNA affected viral production in A549 cells. The core networks show representative molecules related to three main type I IFN signaling pathways. Knockdown of target gene with siRNA in PR8-infected cells increased (red circles), decreased or did not change (black circles) PR8 virus production compared with control siRNA. Each assay was performed three times in independent experiments. (B) The Venn diagram indicates the number of genes overlapping and unique in TLR3/TICAM, TLR7/MyD88 and RIG-I/IPS-1 pathway. The 23 genes were selected from the first screening and most target genes were grouped in RIG-I/IPS-1 pathway.

To understand which pathway is most important for improvement of viral propagation, we mapped the genes and their products identified in the type I IFN-related main pathways (Fig. 2A) and categorized these molecules into 3 groups (Fig. 2B). As the knockdown of NFKB1 enhanced the production of PR8 virus, we thought that NFKB1 might be one of the candidates. NFKB1 serves in downstream signaling pathways leading to the activation of type I IFN production and a common molecule in the three signaling pathways (Fig. 2A and B). Also, knockdown of TBK1, SMAD4 or IRF7 enhanced the production of PR8 virus (Figs. 2A and S1). Seventeen out of 23 genes were shown to be critically related to RIG-I/IPS-1-mediated type I IFN production (Fig. 2B). These results indicate that the genes mainly involved in RIG-I/IPS-1 signaling pathway enhance the production of PR8 virus in A549 cells when knocked down by siRNA. Regarding the molecules upstream of IRF7, knockdown of DDX58 (also known as RIG-I) or MyD88 did not affect the viral production (Figs. 2A and S1). It is interesting to note that knockdown of IRF3 did not affect the production of PR8 virus. However, knockdown of NOD2, MAVS (also known as IPS-1, VISA or Cardif) or IRF7 significantly enhanced the production of PR8 virus (Figs. 2A and S1). Of these highly potential candidates, IRF7 was the most promising molecule because knockdown of IRF7 showed the greatest enhancement of PR8 virus production in A549 cells (data not shown). Thus, we examined whether the same thing holds true for MDCK cells.

### Knockdown of IRF7 Enhances the Production of PR8 Virus in MDCK Cells

To identify a specific gene(s) in canine MDCK cells, we performed second screening of a set of 9 genes involved in RIG-I/IPS-1 signaling pathway for enhancement of virus production in MDCK cells by siRNA-mediated knockdown. Since the sequences for IFN-related genes of canine MDCK cells are not available, we first performed RT-PCR of mRNA for target genes using specific primers designed based on GenBank or Ensemble sequence database. Then, we determined the entire or partial coding sequences of each gene and designed siRNAs for each target gene in MDCK cells. A number of genes showed some sequence variations from the reference sequence (data not shown). Regarding IRF7, canine IRF7 (Transcript ID: ENSCAFT00000010569 in Ensembl data base) was identified as the most similar gene to human IRF7 by BLAST. The RT-PCR product of IRF7 gene in MDCK cells showed a single band with the amplicon size (855 bp) expected from the database of canis lupus familiaris genome and had almost identical nucleotide sequences to those included in the database. However, the sequence of the part encoding N-terminal portion of IRF7 with length of about 70 nucleotides was difficult to determine. Based on the obtained sequences, we designed siRNAs targeting three different sites at nucleotide 454, 449 and 380 in the coding region of IRF7.

**Figure 3:**
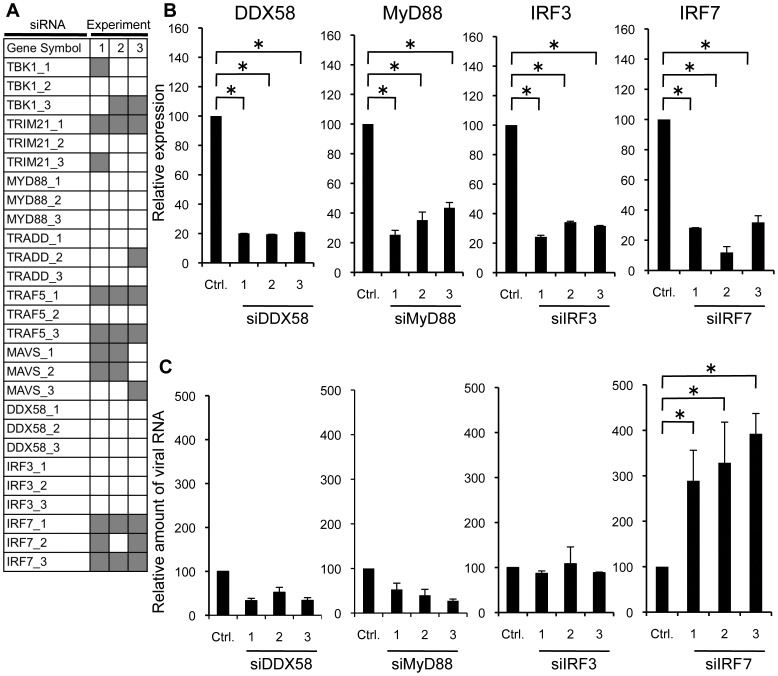
Knockdown of IRF7 enhances PR8 virus production in MDCK cells. (A) MDCK cells were transfected with siRNAs targeting the indicated genes. At 48 h post-transfection, the cells were infected with PR8 virus at a MOI of 0.01. After 24 h of incubation, viral RNA in culture supernatant was measured by quantitative real-time RT-PCR. The gray boxes in the panel mean that siRNA which targets indicated gene showed more than 2-fold enhancement of the virus production compared with negative control siRNA. (B) Total RNA from cells was extracted to monitor knockdown efficiency of target gene in the cells. The endogenous expression of target genes was measured by quantitative real-time RT-PCR with SYBR green. (C) MDCK cells were transfected with three siRNAs targeting genes or negative control siRNA. At 48 h post-transfection, the cells were infected with PR8 virus at a MOI of 0.01. After 24 h of incubation, the RNA from culture supernatant was isolated to examine the amounts of a specific viral RNA. The data are representative results of three independent experiments. Asterisks indicate statistically significant differences compared with the control (*P<0.05).

All designed siRNAs were introduced into MDCK cells separately and the efficiency of virus production was monitored (Fig. 3A). As in A549 cells, the siRNA which enhanced virus production more than 2-fold compared with control was regarded positive (shown as a gray box in Fig. 3A). Inhibition of IRF7, MAVS, and TRAF5 by siRNA-mediated knockdown reproducibly increased virus production. Particularly, IRF7 showed the highest reproducibility amongst the three candidate genes. Therefore, we focused on the IRF7 for further validation. As shown in Fig. 3B, introduction of siRNA for IRF7 reduced the endogenous expression of this gene (90–70% reduction), and the viral titer showed about 4-fold increase in the culture supernatant of cells transfected with specific siRNAs targeting IRF7 (Fig. 3C). As in A549 cells, we also examined whether knockdown of IRF3, MyD88 or DDX58 affects the efficiency of the viral production in MDCK cells for comparison with IRF7. Knockdown of IRF3, MyD88 and DDX58 by siRNAs was confirmed (Fig. 3B), but did not affect the viral production in MDCK cells (Fig. 3C). In summary, these results indicate that IRF7 inhibits the multiplication of PR8 virus and knockdown of this gene enhances virus production in MDCK cells.

### MDCK Cells Stably Expressing shRNA for IRF7 Show Enhancement of Influenza Virus Production

**Figure 4:**
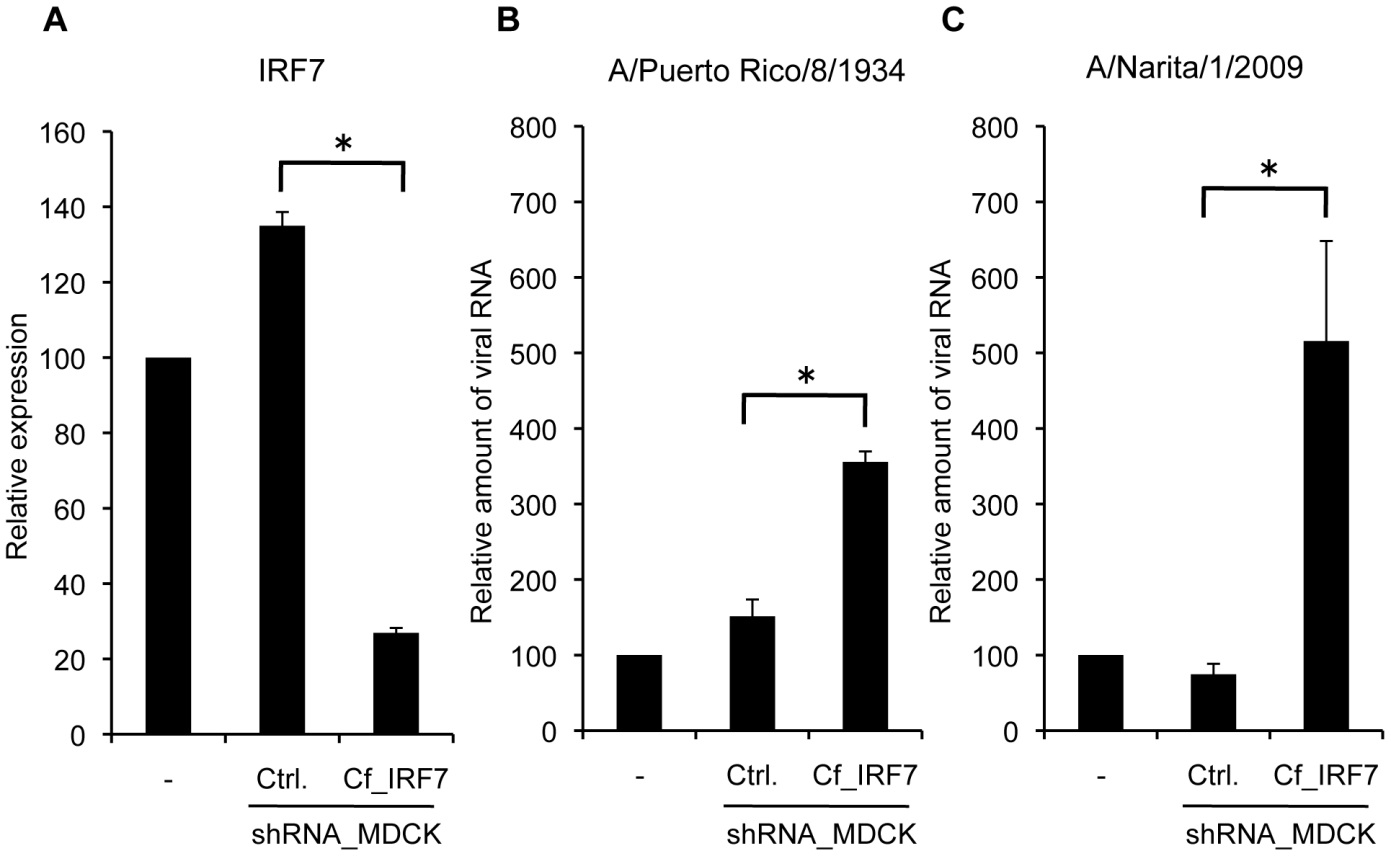
MDCK cells stably expressing shRNA for IRF7 enhances influenza A virus production. (A) The total RNA from parental or transduced MDCK cells was isolated and subjected to quantitative real-time RT-PCR. The knockdown efficiency of IRF7 mRNA expression level by transduced shRNA showed an 80% reduction. (B). The MDCK cells were infected with PR8 virus at a MOI of 0.0003. On day 3 after PR8 infection, the amount of viral RNA in culture supernatant was determined by quantitative real-time RT-PCR. (C) The MDCK cells were infected with A/Narita/1/2009 (A (H1N1) pdm09) virus at a MOI of 0.0003. On day 3 after virus infection, the amount of viral RNA in culture supernatant was determined by quantitative real-time RT-PCR. The relative viral RNA levels were normalized to values for 18S rRNA which was included in carrier RNA, and expressed as the relative (n-fold) value to the level of RNA from control. The data are representative results of three independent experiments. Asterisks indicate statistically significant differences compared with the control (*P<0.05).

To examine whether stable knockdown of IRF7 enhances the production of influenza A virus in MDCK cells, we established MDCK-based cells, which were stably expressing short hairpin RNA (shRNA) for IRF7 by lentiviral transduction. To know the knockdown efficiency, endogenous mRNA of IRF7 was examined by quantitative real-time RT-PCR. The MDCK cells transduced with shIRF7 vector (MDCK-shIRF7) showed an 80% reduction in the transcription of IRF7 compared with the cells transduced with shControl vector (MDCK-shCtrl) (Fig. 4A). The MDCK cells expressing shRNA for either IRF7 or control have the same morphological and physiological characteristics as parental MDCK cells. The cell viability of the parental and the transduced MDCK cells expressing each shRNA showed at least 90% throughout the culture period. To determine the viral growth curve in transduced MDCK cells and parental MDCK cells, the cells were infected with PR8 virus at a MOI of 0.0003 for 1 h. After washing extensively, the cells were incubated for 72 h and the viral RNA in the culture supernatant was measured by real-time RT-PCR. MDCK-shIRF7 cells showed about 3-fold increase in titer of PR8 virus compared with MDCK-shCtrl cells (Fig. 4B).

We also examined the efficiency of propagation of A/Narita/1/2009 (NR1; A(H1N1)pdm09) virus in the modified MDCK cells by real-time RT-PCR. The amount of viral RNA from the culture supernatant of MDCK-shIRF7 cells was 5-fold greater than that from MDCK-shCtrl cells at 3 days post-infection (Fig. 4C).

To confirm that more viral antigen is actually produced from MDCK cells expressing shRNA for IRF7, we performed hemagglutination (HA) assays for quantification of H1N1 viruses. The HA titers of PR8 and NR1 produced from MDCK-shIRF7 cells was twice to 8 times higher than those from MDCK-shCtrl cells (Table 3). To verify that production of hemagglutinin of A(H3N2) virus and B virus is enhanced in MDCK-shIRF7 cells, we performed HA assays for quantification of A/Victoria/361/2011 (VC361; H3N2) and B/Florida/4/2006 (FL4; type B, Yamagata lineage) produced from modified MDCK cells. The HA titers of VC361 and FL4 from MDCK-shIRF7 cells were 4 to 8 times higher than those from MDCK-shCtrl cells (Table 3). These results indicate that the production of influenza viruses is significantly enhanced in the MDCK cells with lower expression level of IRF7.

**Table 3:** HA titers of influenza viruses produced from modified MDCK cells.

Experiment No.	Influenza virus	Subtype/Lineage	sh-Control	sh-IRF7	Input virustiter/well	Time after infection
#1	A/Puerto Rico/8/1934	H1N1	8	16	100 pfu	48 h
	A/Narita/1/2009	H1N1pdm09	4	16	1000 TCID_50_	96 h
	A/Victoria/361/2011	H3N2	4	16	100 TCID_50_	72 h
	B/Florida/4/2006	Yamagata	4	16	100 pfu	72 h
#2	A/Puerto Rico/8/1934	H1N1	8	16	100 pfu	48 h
	A/Narita/1/2009	H1N1pdm09	8	32	1000 TCID_50_	96 h
	A/Victoria/361/2011	H3N2	4	32	100 TCID_50_	72 h
	B/Florida/4/2006	Yamagata	8	32	100 pfu	72 h

### Enhancement of Viral Propagation by siRNA for IRF7 is not Associated with Inhibition of Type I IFN Induction

**Figure 5:**
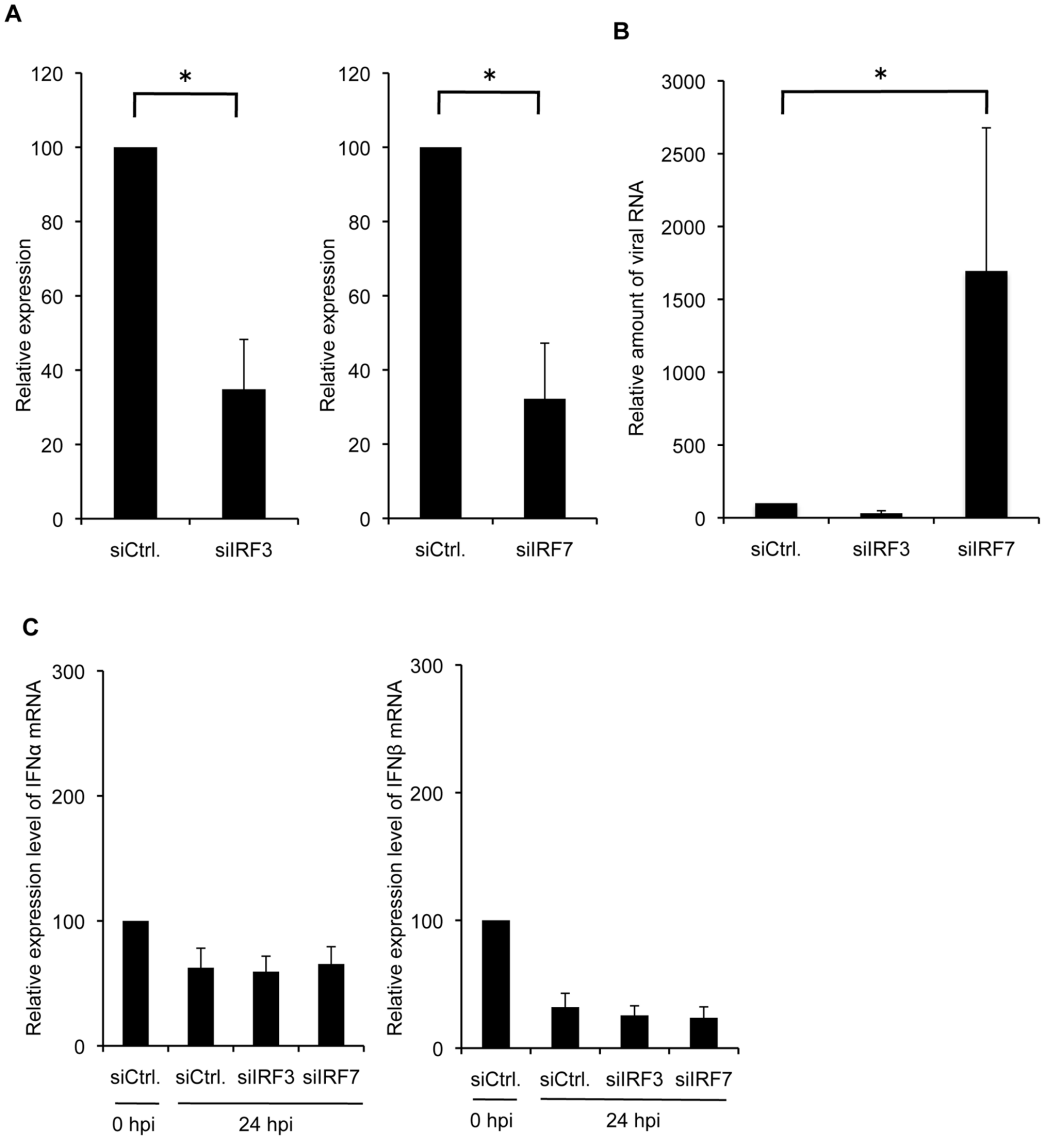
Enhancement of viral propagation with siIRF7 is not mediated by inhibition of type I IFN induction. (A) The total RNA from siRNA-transfected MDCK cells was isolated and subjected to quantitative real-time RT-PCR. (B) The siRNA transfected MDCK cells were infected with PR8 virus at a MOI of 0.03. The amount of viral RNA in culture supernatant was determined by quantitative real-time RT-PCR. Data are shown as fold change in amounts of viral RNA from siRNA-transfected cells compared with that from control cells. (C) The expression of mRNA for each IFN-α/β was measured at indicated time points after infection by quantitative real-time RT-PCR. Data are shown as fold change in expression of mRNA for each IFN-α/β compared with that of control siRNA-transfected cells and normalized to the values for 18S rRNA. The data are representative results of three independent experiments. Asterisks indicate statistically significant differences compared with the control (*P<0.05).

Since knockdown of IRF7 enhanced PR8 virus production in infected MDCK cells, we next examined whether this enhanced viral production by siRNA for IRF7 might be due to the reduced expression of IFN-α/β. To investigate the effects of knockdown of IRF7 on expression levels of IFN-α/β in virus-infected MDCK cells, siRNA for IRF3 or IRF7 was transfected into MDCK cells and the cells were infected with PR8 virus. The efficient knockdown of IRF3 or IRF7 mRNA (70% reduction) was confirmed by quantitative real-time RT-PCR at 48 h post-transfection (Fig. 5A). The viral RNA from culture supernatant and total RNA from cell lysate were extracted at 24 h post-infection (hpi). The relative expression level of endogenous IFN-α/β was quantified by real-time RT-PCR. Notably, while knockdown of IRF7 significantly increased PR8 virus production by about 9-fold compared with the control at 24 hpi (Fig. 5B), the level of IFN-α/β induced in the infected MDCK cells with siIRF7 was not significantly impaired compared with those in MDCK cells with control siRNA (Fig. 5C). On the other hand, knockdown of IRF3 did not affect the virus production (Fig. 5B) and the expression levels of IFN-α/β remained unchanged after infection of siIRF3-transfected MDCK cells (Fig. 5C). IFN-α/β proteins in the supernatants, which were quantified by ELISA (PBL Biomedical Laboratories, Piscataway, NJ), were below the detectable level (<12.5 pg/ml) at 24 hpi in infected-A549 cells (data not shown). Collectively, these results suggest that knockdown of IRF7 enhances PR8 virus production by an unknown mechanism including pathways independent of IFN-α/β.

## Discussion

Influenza virus is currently being produced in embryonated hen’s eggs, but a well-defined cell line is considered to have merit for production of influenza virus to address concerns about the limitations of egg-based influenza vaccines. Potential advantages of cell-culture technology over conventional egg-based technology are as follows: elimination of the long lead time required for egg-based production systems, ease of supply of a substrate that is not susceptible to virulent virus strains, a more controlled production process involving closed-system bioreactors with reduced risk of microbial contamination, and the isolation and replication of influenza viruses without significant egg passage-dependent antigenic changes [4].

The aim of this study is to establish engineered MDCK cells that support efficient influenza virus propagation for better vaccine production. Because inhibition of IFN signaling seems to be a simple and efficient way to increase viral yield [16]–[18], we first screened, to identify target genes for such inhibition, a series of siRNAs for 78 human type I IFN-associated cellular genes (Fig. S1). We used A549 human lung adenocarcinoma cell line for the first screening and MDCK cells for the second screening because human genome database has much more information than canine genome database and pre-designed siRNA libraries are available for human genes but not for canine genes. By siRNA-mediated knockdown, a number of genes that are involved in the RIG-I/IPS-1 signaling pathway enhanced the production of PR8 virus in A549 and MDCK cells (Figs. 2 and 3). From the screening experiments, we identified the human IRF7 gene in A549 cells and a canine IRF7 in MDCK cells as a target gene for the knockdown-induced enhancement of influenza A virus propagation (Figs. 2, 3, 4 and S1). IRF7 seems to be reasonable as a gene to enhance virus propagation when knocked down by siRNA because it is located at the most downstream of the pathways for transcriptional activation of type I IFN and it is the common gene in three major signaling pathways.

To confirm that stable knockdown of IRF7 in MDCK cells enhances production of influenza viruses, we established MDCK cells expressing shRNA for IRF7. By quantitative RT-PCR, MDCK-shIRF7 cells were shown to produce more PR8 viruses than MDCK-shCtrl cells (Fig. 4). HA assays for PR8 and other viruses including H3N2 and B viruses demonstrated that more viral antigens were produced from MDCK-shIRF7 cells than from MDCK-shCtrl cells (Table 3). These results suggest that MDCK-shIRF7 cells can propagate a variety of influenza viruses more efficiently than MDCK-shCtrl cells.

In addition to IRF7, knockdown of MAVS, but not MyD88 or DDX58, enhanced the production of PR8 virus from the siRNA-transfected cells (Figs. 3A and S1). Knockdown of NOD2 also exhibited enhancement of PR8 virus production (data not shown). Sabbah et. al. demonstrated that NOD-like receptors such as NOD2 could act as a PRR for RSV and influenza A virus [19]. Therefore, we speculate that there may be a reciprocal cross talk between NOD2 and MAVS signaling pathways.

In contrast to IRF7, knockdown of IRF3 did not affect the viral production (Figs. 3C and 5B) even though IRF3 and IRF7 are considered to play essential roles in innate immune responses to virus infections. Of the IRF family, IRF7 plays an important role in anti-viral responses and can be activated in a similar manner to IRF3 during viral infection [20], [21]. IRF7 is largely responsible for IFN production in response to viral infection, as evidenced by the abrogation of IFN production in *Irf7* −/− mice, but not in *Irf3* −/− mice [22]. Importantly, although the virus production was significantly enhanced in siIRF7 cells compared with siCtrl cells and siIRF3 cells at 24 hpi (Fig. 5B), there was no statistically significant difference (P>0.05) in the expression levels of IFN-α/β mRNA among cells transfected with siRNA for Ctrl, IRF3 and IRF7 under the conditions described (Fig. 5C). These results suggest that RNAi-mediated knockdown of IRF7 in MDCK cells enhances virus propagation through an unknown process that includes mechanisms other than inhibition of IFN-α/β induction.

Many viruses have evolved mechanisms to escape the IFN system, an antiviral defense of host cells [23], [24]. Influenza A viruses have at least 3 viral proteins which counteract the IFN signaling: NS1, PB1-F2 and PB2. NS1 binds directly to Cleavage and Polyadenylation Specificity Factor 30 (CPSF30) and inhibits maturation of IFN, and other cellular mRNAs in the nucleus [25]. NS1 can associate with RIG-I, as well as TRIM25, a ubiquitin ligase required for RIG-I activation, to prevent its downstream activation of the IFN-β promoter [9], [26]–[28]. Both IRF3 translocation and NFκB activation are impaired in the presence of NS1, which blocks the induction of pro-inflammatory cytokines and IFNs [29], [30]. PB2, a subunit of the influenza virus RNA polymerase, interacts with MAVS and inhibits IFN-β expression [31]. The viral PB1-F2 inhibits RIG-I mediated induction of IFN by suppressing MAVS function [32]. Induction of IFN-α/β was not observed in MDCK cells by 24 h after infection with PR8 virus (Fig. 5C). This may be due to functions of viral proteins which block production of IFN-β. In our screening of siRNA library for human and canine genes, knockdown of RIG-I showed no significant increase in propagation of influenza virus (Figs. 3A and S1. RIG-I is shown as DDX-58 in these figures.). It is possible to consider that in both siCtrl-transfected cells and siRIG-I-transfected cells, the function of RIG-I was strongly blocked by viral proteins. It seems reasonable to assume that when the function of some cellular gene product is sufficiently inhibited by virus-encoded protein(s) unrelated to siRNA, additional inhibition of its function by siRNA may not show a significantly different phenotype. Inhibition of IFN signaling by viral proteins would explain, at least in part, the reason why knockdown of some IFN-related genes did not show enhancement of virus replication. Furthermore, it could also be considered that the IFN-related genes, that show enhancement of virus propagation triggered by siRNA-mediated knockdown, may have another function different from and independent of the IFN signaling so far described. Considering the observation in the present paper, this could be consistent with the idea that the enhancement of influenza virus propagation by siRNA for IRF7 is caused by an unknown cellular process that includes mechanisms other than inhibition of IFN-α/β induction.

Cell-based manufacturing has many potential merits over egg-based manufacturing, but the major drawback of cell-based technique at present may be cost. The cost of cell-based vaccine would be high due to the scale required to generate sufficient products. If yield of viral antigen from cell substrate is improved, it will contribute to reduction of the culture scale and the cost for vaccine production. We consider that our modified MDCK cells could be one of the potential remedies for the shortcoming of cell-based manufacturing. Increase in virus yield by manipulation of cell substrates would turn mammalian cell manufacturing into a more viable approach.

Here, our results demonstrate that the novel MDCK cells expressing shRNA for IRF7 can produce twice to 8 times more influenza viruses than control cells and they will be useful for larger and more rapid production of influenza vaccines. Furthermore, these cells will have an advantage for isolating influenza viruses in a small amount in clinical specimens and thus preparing seed viruses for vaccine production. The novel MDCK cells could contribute to expanding availability of influenza vaccine to a large number of people worldwide.

## Supporting Information

Figure S1 **Screening of siRNA library for human typeI interferon-related genes.** A549 cells were transfected with siRNAs targeting 78 different genes at a final concentration of 10 nM. At 48 h post-transfection, A549 cells were infected with PR8 virus at a MOI of 0.01. At 24 hpi, the viral RNA from culture supernatant was extracted for quantitative real-time RT-PCR. The relative amount of viral RNA was normalized to the values for 18S rRNA which was included in carrier RNA. The gray box means that siRNA targeting the indicated gene increases the amount of viral RNA more than 2-fold compared with control siRNA in A549 cells. Each assay was performed three times in independent experiments.
(TIF)
