# Supplementary figures and images for: High Yield Production of Influenza Virus in Madin Darby Canine Kidney (MDCK) Cells with Stable Knockdown of IRF7
Source: PLoS One. 2013 Mar 26;8(3):e59892. doi: 10.1371/journal.pone.0059892 (PMC3608535; doi:10.1371/journal.pone.0059892)

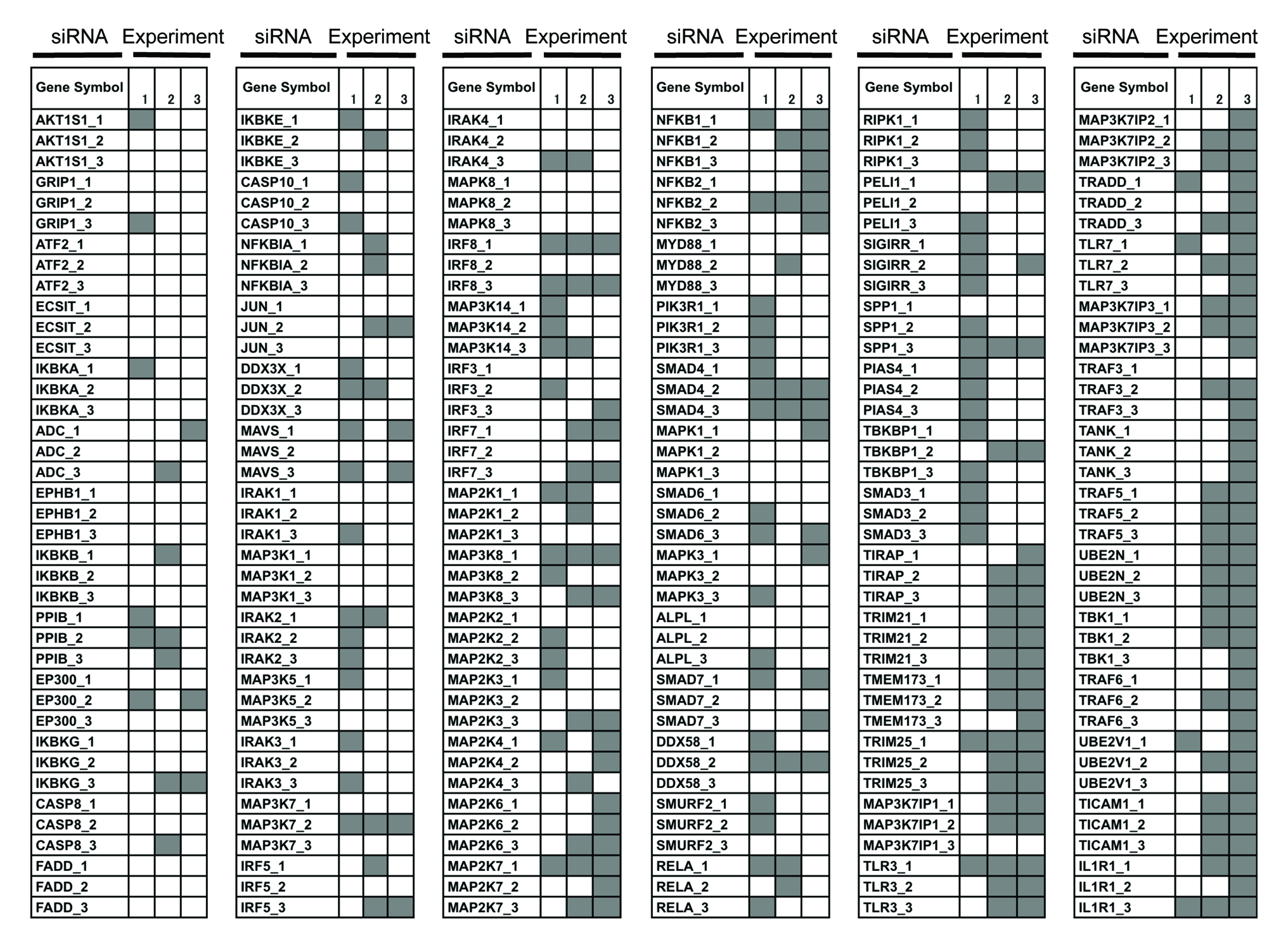

Supplement: Figure S1 — Screening of siRNA library for human typeI interferon-related genes. A549 cells were transfected with siRNAs targeting 78 different genes at a final concentration of 10 nM. At 48 h post-transfection, A549 cells were infected with PR8 virus at a MOI of 0.01. At 24 hpi, the viral RNA from culture supernatant was extracted for quantitative real-time RT-PCR. The relative amount of viral RNA was normalized to the values for 18S rRNA which was included in carrier RNA. The gray box means that siRNA targeting the indicated gene increases the amount of viral RNA more than 2-fold compared with control siRNA in A549 cells. Each assay was performed three times in independent experiments. (TIF) [file pone.0059892.s001.tif]
